# Gender differences in the prospective association between maternal alcohol consumption trajectories and young adult offspring’s problem gambling at 30 years

**DOI:** 10.1186/s40405-016-0010-3

**Published:** 2016-04-08

**Authors:** Nam T. Tran, Alexandra Clavarino, Gail Williams, Jake M. Najman

**Affiliations:** 1 School of Social Science, University of Queensland, St Lucia, Brisbane, QLD 4072 Australia; 2 Department of Sociology, Academy of Journalism and Communication, Hanoi, Vietnam; 3 School of Pharmacy, University of Queensland, Brisbane, Australia; 4 School of Public Health, University of Queensland, Brisbane, Australia; 5 Queensland Alcohol and Drug Research and Education Centre, Brisbane, Australia

**Keywords:** Problem gambling, Young adult, Alcohol consumption, Trajectory, Gender, Longitudinal study, Australia

## Abstract

Although a large number of studies have examined the association between young adult’s alcohol consumption and their problem gambling behaviours, none of these studies address the prospective association between mother’s alcohol consumption and their young adult offspring’s problem gambling behaviours. Using data from a 30 year prospective pre-birth cohort study in Brisbane, Australia (n = 1691), our study examines whether different maternal alcohol consumption trajectories predict offspring’s risk of problem gambling behaviours and whether these associations differ by the young adults’ gender. Offspring’s level of problem gambling behaviours was assessed by the short version of the Canadian Problem Gambling Index, with about 10.6 % of young adults having some risk of problem gambling behaviours. Trajectories of maternal alcohol consumption were determined by group-based trajectory modelling over five time points. Our study found that mother’s alcohol consumption pattern fits into three drinking trajectory groups, namely abstainers (17.2 %), a low-stable drinkers group (64.6 %) and a moderate-escalating drinkers group (18.2 %). Multivariate logistic regression analyses showed that the moderate-escalating alcohol trajectory group is independently associated with a risk of their male young adult offspring having problem gambling behaviours at 30 years—even after adjustment for a range of potential confounding variables. Mothers who exhibit a persistent life course pattern of moderate-escalating drinking have male children who have a high risk of engaging in problem gambling behaviours. Offspring’s alcohol consumption partially mediated the association between maternal drinking trajectories and young adult’s risk of problem behaviours. High levels of maternal alcohol consumption may lead to male offspring antisocial behaviours. Programs intended to address problem gambling behaviours by young adults may need to focus on male group with a focus which specifically addresses family influences as these contribute to gambling behaviour.

## Background

Between 5.3 and 12.1 % of Australian adults are estimated to manifest behaviours associated with problem gambling (Acil Allen Consulting 2014; QDJAG 2012; Billi et al. 2014; The Social Research Centre 2013; Davidson and Rodgers 2010; Sproston et al. 2012). These proportions are generally consistent with figures reported for other countries such as the United States, Canada, Sweden, and the United Kingdom (Abbott et al. 2014; Orford et al. 2012; Wardle et al. 2011; Williams et al. 2012). Studies across countries have shown that prevalence estimates for problem gambling among adolescents and young adults are higher than in adult populations (Delfabbro et al. 2014; Scholes-Balog et al. 2014; Volberg et al. 2010). Among young people aged 13–17 years, between 60 and 80 % gamble at least once per year, and around 3–5 % report symptoms of problem gambling or pathological gambling (Delfabbro et al. 2014).

A range of factors have been associated with the risk of problem gambling among adolescents and young adults, such as gender, age, antisocial behaviours, academic performance, family socioeconomic status, parental gambling involvement (Barnes et al. 2005; Delfabbro et al. 2014; Forrest and McHale 2012; Shead et al. 2010; Williams et al. 2015), as well as alcohol consumption. It is not only the extent to which alcohol consumption normally coexists with gambling behaviours; it may also be an antecedent of problem gambling, suggesting considerable implications for intervention strategies. The correlation between alcohol consumption or alcohol use disorder and problem gambling among adolescents and young adults has been well documented in cross-sectional studies (Banwell et al. 2006; Delfabbro 2008; LaBrie et al. 2003; Nehlin et al. 2013; Welte et al. 2001). For example, a national survey of gambling among 10,765 college students in the United States found that students who gambled were less likely to abstain from drinking alcohol (LaBrie et al. 2003); and a cross-sectional Swedish survey of youth aged 16–24 years found that, among males, the higher the alcohol consumption, the greater the likelihood of gambling and problem gambling behaviours (Fröberg et al. 2013). The few longitudinal studies examining alcohol consumption by adolescents as a risk factor for problem gambling consistently show that higher levels of alcohol consumption or early initiation of alcohol use have been associated with problem gambling (Abbott et al. 2004b; Barnes et al. 2002; Goudriaan et al. 2009; Scholes-Balog et al. 2014; Walker et al. 2012). We have previously found that those who started their drinking alcohol before age 15 years were more likely to be gamblers than those who did not drink (Hayatbakhsh et al. 2013).

The gambling literature provides cross-sectional and longitudinal evidence of the association between adolescents’ alcohol consumption and problem gambling behaviours; however, to our knowledge, there is no study examining the relation between maternal alcohol consumption and their young adult offspring having problem gambling behaviours. Comorbidity between problem gambling and other substance use, including alcohol consumption or alcohol use disorder, has long been recognised (Delfabbro 2008; Lorains et al. 2011; Scholes-Balog et al. 2014). These studies suggest that those who consume or binge on alcohol are more likely to gamble and to have problem gambling behaviours.

Further, previous research has provided evidence showing the association between parental alcohol consumption and offspring’s outcomes such as alcohol use and other substance use, or child antisocial behaviours (Cleveland et al. 2014; Englund et al. 2008b; Mares et al. 2011; Van Der Vorst et al. 2009; Yule et al. 2013). Researchers argued that if parents drink at heavy level, they are less supportive and more aggressive toward their children. They also pay less attention to their child, and are less inclined, or less able to monitor their child’s behaviour (Alati et al. 2010; Handley and Chassin 2013; Piko and Balázs 2012; Van Zundert et al. 2006). In this study, we expected that there will be an association between maternal alcohol consumption and young adults’ problem gambling behaviour. A longitudinal association of maternal alcohol consumption predicting offspring gambling behaviour has not previously been reported. Such research would provide a basis for determining whether a mother’s alcohol consumption trajectory is associated with her adolescent’s problem gambling behaviours. This may help in the development of prevention programs for both alcohol consumption and problem gambling.

There are numerous studies investigating socioeconomic status and other factors such as paternal alcohol consumption as these are related to offspring’s alcohol consumption and gambling problem (Billi et al. 2014; Fröberg et al. 2015; Mares et al. 2011; Melotti et al. 2013). For example, male adolescents are more likely to be involved in problem gambling than female adolescents (Abbott et al. 2014; Delfabbro 2008; Scholes-Balog et al. 2014); maternal alcohol consumption has a greater affect on male drinking than female drinking (Cleveland et al. 2014; Englund et al. 2008a), lowest social economic status women might be heaviest drinkers and have children who are more likely to have a drinking problem (Cerdá et al. 2011; Melotti et al. 2013); or parental substance use problems have an adverse impact on offspring’s alcohol consumption (Biederman et al. 2000; Mares et al. 2011). These factors possibly confound and mediate the association between maternal alcohol consumption and offspring’s outcomes. We included mother and child’s socioeconomic status as confounders; while paternal alcohol consumption was treated as a mediating variable for the purpose of this study.

The present study uses prospective data from the Mater-University of Queensland Study of Pregnancy (MUSP), comprising a linked pre-birth cohort of mothers and children spanning over 30 years to investigate the association between maternal alcohol consumption trajectories and offspring’s problem gambling controlling for other possible factors. We hypothesise that (1) higher levels of maternal alcohol consumption are associated with young adult’s problem gambling; and (2) mother’s pattern of alcohol consumption has a greater impact on male than female young adult gambling behaviours.

## Methods

### Participants and procedures

Data were taken from the Mater-University of Queensland Study of Pregnancy and its outcomes (MUSP), a prospective pre-birth cohort study of women enrolled at a public obstetric hospital in Brisbane, Australia between 1981 and 1984. Details of the study have been described elsewhere (Najman et al. 2005, 2014). Women were recruited at their first clinic visit (Time 1), at approximately 18 weeks of gestation, and 6753 women were asked about their alcohol consumption, social demographic characteristics, life style behaviours, and health information before pregnancy. These women were re-interviewed when their child was 6 months (Time 2), 5 (Time 3), 14 (Time 4) and 21 (Time 5) years of age. Their offspring were also interviewed for the 14, 21 and 30 year follow-up surveys (Time 4, Time 5, Time 6, respectively). The present study is based on the sub-sample of 1691 mothers and their children remaining in the study at 30 years. The sub-sample includes mothers who provided details of their alcohol consumption for up to five phases of the study (Time 1–5) and their children who provided data on gambling behaviours at 30 years (Time 6). Written informed consent was obtained from mothers at all data collection phases and from the young adults at the 30 year follow-up of the study. Ethics committees from the Mater Hospital and from The University of Queensland approved each phase of study.

### Measures

#### Measure of outcome variable

*Young adult’s risk of problem gambling behaviours.* At 30 year follow-up, young adults were asked the question ‘*Do you spend money on gambling?*’ with possible responses ‘Yes/No’. Respondents had not gambled were not asked the nine CPGI question whereas those responding ‘Yes’ were asked to complete a short version of the Canadian Problem Gambling Index (CPGI) to determine their levels of problem gambling behaviours over the last 12 months. The CPGI has been tested and re-tested for its validity and reliability in both general population and clinical sample surveys with Cronbach’s alpha = 0.84, higher than the one tested for other measures such as Diagnostic and Statistical Manual of Mental Disorder, Fourth Edition (DSM-IV) and South Oaks Gambling Screen (SOGS) (Ferris and Wynne 2001). The tool has been widely used in previous studies in Queensland in the 2001, 2003–2004, 2006–2007, and 2011–2012 (Attorney-General 2012), in other states in Australia and other countries (Abbott et al. 2004a). The short version of CPGI comprises nine questions as follow: (1) Bet more than afford to lose, (2) Gamble with larger amounts, (3) Tried to win back losses, (4) Borrowed or sold to get money, (5) Felt have a problem with gambling, (6) Caused health problems, (7) Told had gambling problem, (8) Caused financial problems, and (9) Felt guilty about gambling.

The response options for the CPGI are “never” which scored 0; “sometimes” scored 1; “most of the time” scored 2; and “almost always” scored 3. In the present study, together with the responses from spending money on gambling, individual scores on the nine questions were added to generate an overall score ranging from 0 to 27 where respondents were classified into one of four gambling behaviour categories: 0 = non problem gamblers (including those who had not gambled and who had gambled for recreational purpose), 1–2 = low risk, 3–7 = a moderate risk, and 8+ = a problem gambler (Ferris and Wynne 2001). Consistent with other studies (Acil Allen Consulting 2014; Billi et al. 2014; Sproston et al. 2012), the proportions of moderate risk and problem gamblers in our study were low, 3.2 and 1.1 %, respectively. In order to increase the statistical power, we collapsed the four problem gambling behaviours into two groups as follows: non problem gambler or no risk behaviour—some risk behaviours of problem gambling (included low risk, moderate risk, and problem gamblers). The distribution of no risk and some risk behaviours of problem gambling is also the cut-off point of 10 % of the population sample.

### Measure of main predictor

The main exposure in this study is the maternal alcohol consumption trajectory over 21 years. These trajectories were based upon self-reported information from mothers at Time 1, Time 2, Time 3, Time 4, and Time 5, 21 years after the birth of their child.

*Maternal alcohol consumptionat each phase of study*. At each survey, mothers were asked how often they drank alcohol and how much alcohol they consumed on each occasion. Respectively, six pre-specified response options ranging from never to daily alcohol consumption and from none to seven or more standard drinks were provided. Guidelines from the Australia National Health and Medical Research Council (NHMRC 2009) suggest that women’s alcohol consumption should be calculated on the basis of weekly alcohol consumption standard drink (containing 10 grams of pure alcohol) scores. In our study, alcohol consumption scores were estimated by a method described by Dawson (2003), whereby the mid-point estimate of frequency was multiplied by the mid-point of quantity consumed.^1^ For consistency with other studies (French et al. 2014; Powers and Young 2008), respondents’ levels of alcohol consumption were categorised as non-drinkers (never drink), occasional drinkers (<1 drink per week), moderate drinkers (from 1 to 14 drinks per week), and heavy drinkers (>14 drinks per week).

*Long term trajectories of maternal alcohol consumption*. Long term trajectories of alcohol consumption by women were examined by using group-based trajectory modelling (Nagin 2005). We used a censored normal model suggested by Jones and Nagin (2012) to examine the changes of alcohol consumption. Consistent with other studies (Bobo et al. 2013; Casswell et al. 2002), square root transformation was applied to alcohol data to reduce the effects of the skewed distribution. First, we used an unconditional model to identify the number of trajectory groups. Then, because the patterns of maternal alcohol consumption over the mothers’ life course may depend on the number of children women may have (Brennan et al. 2010; Roche and Deehan 2002; Tran et al. 2014; Wilsnack 2012), we fitted a conditional model by adding parity variable as a function of time varying covariate in the model while simultaneously estimating the parameters that defined the trajectory group.

The estimation of the unconditional trajectory group model showed that we could identify between two to four drinking trajectory groups in our sample (see more in “Appendix” for details of the Bayesian Information Criterion (BIC), group membership, and the posterior probabilities for all four trajectory models). The three-group model had the largest BIC (−22675.65) with mean posterior probability ranging from 0.86 to 0.93, suggesting that it represented a better fit than did the other models. The fitted model provided distinct drinking trajectories that were much bigger than the minimum average posterior probability of .70 for all groups (Nagin 2005).

In the conditional trajectory model, adding the number of offspring over five time period slightly improved the model fit (BIC = −21068.01). On the basis of BIC criteria, we selected a conditional model with three drinking trajectory groups as our final model for examining the association between maternal alcohol consumption trajectories and their young adult’s risk of problem gambling behaviours.

**Fig. 1:**
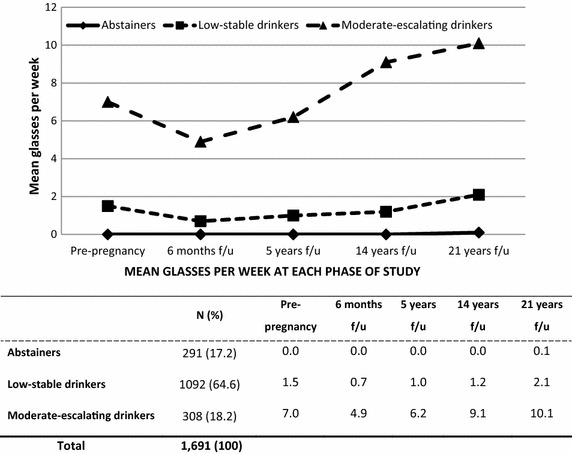
Maternal alcohol consumption trajectories at five times over 21 years

Figure 1 displays the shape of three drinking trajectories and the mean alcohol consumption level of each trajectory over five time points.

- The largest group (an estimated 64.6 % of the sample, average posterior probability of membership = .92) was labelled as the *low-stable drinkers* group. Women in this trajectory group consumed an average of 1.5 standard drinks per week at Time 1. They reduced their consumption by half when they found out they were pregnant, then slowly increased their consumption, but their level of alcohol consumption was still low at Time 3 and Time 4, 14 years after the birth of their baby. After 21 years (Time 5), their weekly average alcohol consumption was the equivalent to two glasses a week.
- The next largest group consisted of *moderate-escalating drinkers* (an estimated 18.2 % of sample, average posterior probability of membership = .92). Members of this group were those who consumed alcohol at a moderate level (7 standard glasses per week) at Time 1, pre-pregnancy. They slightly reduced their consumption at Time 2, but returned to their pre-pregnancy drinking level at Time 3, a 5 year follow-up; then, gradually continued to increase their level of alcohol use. At the 21 year follow-up, the weekly average alcohol consumption for these women was 10.1 standard glasses per week.
- The third group was *the abstainers* (an estimated 17.2 % of sample, average posterior probability of membership = 0.86) who reported having had no alcohol consumption or minimal consumption at the five survey times.

#### Covariates

To examine the independent association between maternal alcohol consumption trajectories over 21 years and their young adult’s risk of problem gambling behaviours, we included a number of potential confounding variables in different models. These covariates included mother’s age and educational level at Time 1, mother’s income and marital status at Time 4; paternal alcohol use problems at Time 4; the young adult’s socioeconomic status (educational levels, income, and marital status) at Time 5, a 21 year follow-up.

In addition, we controlled for offspring’s alcohol consumption which was measured at Time 6, the same time as gambling behaviour is assessed. Offspring’s alcohol consumption was measured using the similar tool (*frequency*-*quantity questions*) assessed mother’s alcohol consumption at each phase of study. Due to the low proportion of heavy alcohol consumption of offspring of mothers who are members of the moderate-escalating drinking trajectory and their risk behaviours of problem gambling, we treated young adult’s alcohol consumption as a covariate to test the possibility that maternal alcohol consumption patterns could be related to offspring’s alcohol consumption which in turn may be associated with offspring’s problem gambling behaviours.

### Analyses of data

The data analyses were performed in four stages. First, we assessed collinearity to examine the correlations among the predictor variable and covariates. The variance inflation factor (VIF), ranging from 1.02 to 1.48, indicated that no collinearity was involved. All predicting variables were included in further analyses.

Second, we examined the association between maternal patterns of alcohol consumption and their young adult offspring’s risk of problem gambling behaviours using logistic regression models. Initially, we examined the crude association between the outcome variable (risk of problem gambling behaviours) and the main predictor (trajectories of maternal alcohol consumption). We subsequently adjusted the association using a range of covariates in different models to examine whether or not each predicting variable/group confounded the association as suggested by previous research (Delfabbro et al. 2014; Forrest and McHale 2012; Hayatbakhsh et al. 2013). We adjusted for mother’s age and educational level at baseline; mother’s income and marital status at the 14 year follow-up (Model 1); then included paternal alcohol related problems at 14 year follow-ups (Model 2); young adult’s socioeconomic characteristics at the 21 year follow-up (Model 3); and young adult’s alcohol consumption at 30 year follow-up (Model 4).

Next, to examine whether the effect of maternal trajectories of alcohol consumption over 21 years on young adult offspring’s risk of problem gambling behaviours varies by the sex of a child, we performed all the analyses separately for male and female young adults.

Of the cohort of 3416 mother–child pairs at Time 6, about 1691 (49.5 %) children were retained and provided information on problem gambling behaviours. We assessed how attrition may have affected our results using a multivariable logistic regression model of loss- to follow-up with young adult’s demographic characteristics at 21 year follow-up (e.g. gender, employment, income, marital status, and impulsive behaviours). Starting from a “missing at random” assumption (Sterne et al. 2009), we used Stata to multiply impute missing data from the main predictor, covariates, and outcome. Initially, we used 20 cycles of imputation for the analyses of imputed data. Subsequently, sensitivity analysis was employed by repeating the whole process using 50 cycles of imputation.

All analyses were undertaken using STATA version 13.0 where a *p* value of <0.05 was adopted as a threshold for significant results. The no risk gambling behaviour group was selected as the reference category in all our analyses. Data are presented in the results section based on the analyses of 1691 mother–children pairs.

## Results

Overall, 10.6 % (n = 180) of participants at age 30 reported having some risk behaviours of problem gambling, of which males accounted for 58.3 % (n = 105). The prevalence of any risk behaviour of problem gambling among male young adult offspring in our sample is consistent with previous studies (Forrest and McHale 2012; Scholes-Balog et al. 2014). Among those who were determined as having some risk of problem gambling behaviours, more than 80 % had a high income and were single; and nearly two-thirds finished their secondary schooling. The univariate analysis between maternal alcohol consumption trajectories and their young adult’s risk of problem gambling behaviours shows that young adult offspring who have mothers in the moderate-escalating group tend to have greater risk of problem gambling behaviours than those with mothers in the low-stable and abstainers groups (15.9 vs 9.7 and 8.5 %, respectively).

Table 1 presents bivariate and multivariate associations between young adult’s risk of problem gambling behaviours and maternal alcohol consumption trajectories. Bivariate results from logistic regression analysis show that the maternal moderate-escalating group is associated with their young adult’s risk of problem gambling behaviours, while the low-stable group is not related to any risk of problem gambling behaviours. The results remained statistically significant for the moderate-escalating group when adjusted for mother and young adult’s socioeconomic status and paternal alcohol problems (model 1–3). The model was not statistically significant after adjustment for offspring’s alcohol consumption, suggesting that the association between mother’s moderate-escalating drinking trajectory and young adult’s risk of problem gambling behaviours was partially mediated by alcohol use by offspring.

**Table 1:** Maternal alcohol consumption trajectories and young adult’s risk of problem gambling behaviours at 30 year follow-up

Maternal alcohol consumption trajectories^a^	No (%)	No risk behaviour^b^	Risk of problem gambling behaviours				
			Odds ratio (95 % CI)				
			Unadjusted	Adjusted			
				Model 1^c^	Model 2^d^	Model 3^e^	Model 4^f^
Abstainers	291 (17.2)	1.0	1.0	1.0	1.0	1.0	1.0
Low-stable drinkers	1092 (64.6)	1.0	1.2 (0.7–1.9)	1.2 (0.7–1.9)	1.1 (0.6–1.9)	1.1 (0.6–2.0)	0.9 (0.5–1.7)
Moderate-escalating drinkers	308 (18.2)	1.0	*2.1 (1.3–3.6)*	*2.0 (1.2–3.5)*	*2.1 (1.1–4.0)*	*2.1 (1.1–4.2)*	*1.6 (0.8- 3.2)*

Italic = *p* < 0.05

^a^Assessed over 21 year follow-up

^b^Ref. No risk behaviours of problem gambling

^c^Controlled for mother’s SES (maternal age and educational levels at Time 1, maternal marital status and income at 14 year)

^d^Controlled for model 1 plus paternal alcohol problems at 14 year

^e^Controlled for model 2 plus young adult’s SES (income, education, marital status) at 21 year

^f^Controlled for model 3 plus young adult’s alcohol consumption at 30 years

 Tables 2 and 3 show the results for male and female young adult offspring separately. Maternal alcohol consumption trajectories did not significantly predict risk of problem gambling behaviours for young adult females (Table 2). However, the analyses among male offspring indicate a strong association between the maternal moderate-escalating group and male adolescents’ risk of problem gambling behaviours with ORs ranging from 3.6 (95 % CI 1.7–7.6) for unadjusted to 4.6 (95 % CI 1.6–12.8) for adjusted analyses (Table 3). Adjustment for offspring’s alcohol consumption at 30 year appeared to reduce the strength of the association between maternal moderate-escalating drinking trajectory and young adult male’s risk of problem gambling behaviours, ORs reducing from 4.6 (95 % CI 1.6–12.8) in model 3–3.0 (95 % CI 1.0–8.7) in model 4. Maternal alcohol consumption for the moderate-escalating group appears to predict problem gambling behaviours for their male rather than female young adult offspring. Women who exhibit a persistent life course pattern of heavier alcohol consumption have male children who have a high risk of engaging in problem gambling behaviours.

**Table 2:** Maternal alcohol consumption trajectories and female young adult’s risk of problem gambling behaviours at 30 year follow-up

Maternal alcohol consumption trajectories^a^	No (%)	No risk behaviour^b^	Risk of problem gambling behaviours				
			Odds ratio (95 % CI)				
			Unadjusted	Adjusted			
				Model 1^c^	Model 2^d^	Model 3^e^	Model 4^f^
Abstainers	178 (16.9)	1.0	1.0	1.0	1.0	1.0	1.0
Low-stable drinkers	686 (65.0)	1.0	0.9 (0.5–1.7)	0.8 (0.4–1.6)	0.6 (0.3–1.4)	0.7 (0.3–1.6)	0.6 (0.3–1.3)
Moderate-escalating drinkers	191 (18.1)	1.0	1.2 (0.5–2.6)	1.0 (0.5–2.3)	1.1 (0.5–2.7)	1.2 (0.5–2.9)	1.0 (0.4–2.6)

^a^Assessed over 21 year follow-up

^b^Ref. No risk behaviours of problem gambling

^c^Controlled for mother’s SES (maternal age and educational levels at Time 1, maternal marital status and income at 14 year)

^d^Controlled for model 1 plus paternal alcohol problems at 14 year

^e^Controlled for model 2 plus young adult’s SES (income, education, marital status) at 21 year

^f^Controlled for model 3 plus young adult’s alcohol consumption at 30 years

**Table 3:** Maternal alcohol consumption trajectories and male young adult’s risk of problem gambling behaviours at 30 year follow-up

Maternal alcohol consumption trajectories^a^	No (%)	No risk behaviour^b^	Risk of problem gambling behaviours				
			Odds ratio (95 % CI)				
			Unadjusted	Adjusted			
				Model 1^c^	Model 2^d^	Model 3^e^	Model 4^f^
Abstainers	113 (17.8)	1.0	1.0	1.0	1.0	1.0	1.0
Low-stable drinkers	406 (63.8)	1.0	1.6 (0.8–3.2)	1.7 (0.8–3.4)	2.1 (0.8–5.2)	2.0 (0.8–5.1)	1.6 (0.6–4.1)
Moderate-escalating drinkers	117 (18.4)	1.0	*3.6 (1.7–7.6)*	*3.7 (1.7–8.0)*	*4.6 (1.7–* *12.6)*	*4.6 (1.6–* *12.8)*	*3.0 (1.0–* *8.7)*

Italic = *p* < 0.05

^a^Assessed over 21 year follow-up

^b^Ref. No risk behaviours of problem gambling

^c^Controlled for mother’s SES (maternal age and educational levels at Time 1, maternal marital status and income at 14 year)

^d^Controlled for model 1 plus paternal alcohol problems at 14 year

^e^Controlled for model 2 plus young adult’s SES (income, education, marital status) at 21 year

^f^Controlled for model 3 plus young adult’s alcohol consumption at 30 years

Of the cohort of 3416 mother–child pairs at Time 5, a 21 year follow-up, about 1725 (50.5 %) were lost to follow-up and did not provide information on gambling behaviours at Time 6, a 30 year follow-up. Young adults who dropped out of the study or were excluded from the study due to not providing gambling information were more likely to be male who did not finish their secondary schooling and had higher income levels (Table 4). Repeated analyses which used 20 cycle and 50 cycle multiple imputations showed similar results as the ones in the main analyses of the sample of 1691 mother–child pairs, suggesting that our findings do not reflect selection bias.

**Table 4:** Multivariate attrition analyses predicting those who were lost to follow-up at 30 year

Predicting variables^a^	Odds of being lost to follow-up	
	Unadjusted	Adjusted^b^
	OR (95 % CI)	OR (95 % CI)
Gender		
Male (Ref.)	1.0	1.0
Female	*0.4 (0.4–0.5)*	*0.5 (0.4–0.5)*
Marital status		
Never married (Ref.)	1.0	1.0
Cohabitation	0.9 (0.8–1.1)	1.0 (0.8–1.2)
Married	0.8 (0.5–1.1)	0.9 (0.6–1.3)
Sep-Div-Wid	2.2 (0.9–5.4)	2.4 (0.9–6.5)
Educational level		
Lower secondary school (Ref.)	1.0	1.0
Secondary school	*0.4 (0.3–0.5)*	*0.4 (0.4–0.5)*
College-TAFE/Uni	*0.4 (0.3–0.5)*	*0.4 (0.4–0.5)*
Income		
$160+ per week (Ref.)	1.0	1.0
$0 to $159 per week	*0.7 (0.6–0.8)*	*0.7 (0.6–0.8)*
Impulsive behaviours		
Nomal (Ref.)	1.0	1.0
High	0.9 (0.7–1.2)	0.9 (0.7–1.1)
Maternal alcohol consumption trajectories		
Abstainers (Ref.)	1.0	1.0
Low-stable drinkers	0.8 (0.7–.0)	0.8 (0.7–1.0)
Moderate-escalating drinkers	*0.8 (0. 6–0.9)*	0.8 (0.6–1.0)

Italic = *p* < 0.05

^a^Assessed at 21 year follow-up

^b^Model was adjusted for all factors listed

## Discussion

Using the data from a linked pre-birth cohort of mothers and children extending over 30 years, we examined the association between mother’s alcohol consumption trajectories and their offspring’s risk of problem gambling behaviour, including the extent of gender differences. We found that a maternal trajectory of moderate-escalating alcohol consumption over 21 years is independently associated with a risk of their young adult offspring having problem gambling behaviours at 30 years—even after adjustment for a range of potential confounding variables, with the exception of alcohol use by offspring. Mothers who consume alcohol at a moderate to heavier level over an extend period of their child’s early life course are more likely to have a child at risk of problem gambling.

The association between mother’s alcohol consumption trajectories and problem gambling behaviour by their adolescent offspring differs by their gender. In our study, there is “cross-gender influence” observed for the moderate-escalating drinkers group. Mother’s moderate-escalating drinking trajectory predicts male offspring rather than female counterpart gambling, specially problem gambling. This finding differs somewhat from previous research which indicates that children tend to model their same sex parents’ substance use (Yeh et al. 2006), but it is supported by the work of Cleveland et al. (2014) and Englund et al. (2008a), showing that maternal alcohol consumption is a good predictor of male alcohol use.

Adjustment for mothers and their young adult offspring’s socioeconomic status does not change the magnitude of the association among young adult male group. However, it is of note that adjustment for paternal alcohol-related problems increased the magnitude of the association (OR = 3.6; 95 % CI 1.7–7.6 in unadjusted model; OR = 3.7; 95 % CI 1.7–8.0 in model 1; and OR = 4.6; 95 % CI 1.7–12.6 in model 2), suggesting that paternal alcohol consumption is associated with and contributes to young adults’ problem gambling behaviour. We were unable to test whether the mother’s or father’s alcohol consumption pattern had the greater impact on their adolescent’s problem gambling behaviours as we did not have this information for fathers. However, our analyses suggest that father’s alcohol use partly predicts child problem gambling outcomes at 30 years in a way that paternal alcohol consumption is likely to influence young adult’s alcohol use which in turn may impact on gambling behaviour. Further research should address the patterns of both paternal and maternal alcohol consumption on adolescents’ problem gambling behaviour.

Our study results show that alcohol consumption by offspring partially mediated the association between mother’s moderate-escalating drinking trajectory and young adult’s risk of problem gambling behaviours. After adjustment for offspring’s alcohol consumption, the association of maternal alcohol consumption and offspring gambling was no longer statistically significant. The results suggest that together with maternal alcohol consumption trajectories, alcohol use by offspring is associated with and contributes to gambling behaviours. Alternatively, the present study implies the co-occurrence of alcohol consumption and gambling problems among offspring, suggesting that such behaviours may have a shared antecedent factor.

Our analyses indicated that not only is there a significant co-occurrence between alcohol consumption and problem gambling among the general sample but also that this association is considerably stronger in males rather than females. Prevention and intervention programs need to target males for this reason.

There are number of possible mechanisms explaining why maternal alcohol consumption is associated with gambling behaviour generally and problem gambling behaviour particularly in offspring. First both alcohol use and gambling behaviour may reflect other influences such as family problems, poor parenting possibly associated with being a teenage mother or a history of family poverty (Barnes et al. 2005; Forrest and McHale 2012; Shead et al. 2010). In our analyses we controlled for a number of confounders and found that the association between maternal alcohol use and gambling remained largely unaffected. To the extent that our measures of family dynamics are associated with family problems and related factors, these dynamics are not responsible for the gambling behaviour of offspring. Secondly, alcohol use and gambling behaviour are both forms of sensation seeking and/or risk taking, and may reflect an underlying pattern of antisocial behaviour (Hayatbakhsh et al. 2013; Magoon et al. 2005; Vitaro et al. 2001). It may simply be the case that offspring of mothers who consume alcohol in a persistent manner are more prone to behave in a risky manner, of which offspring drinking and gambling are a part. Alternatively, males are more likely to consume alcohol and to gamble at a risky level, and females are less likely to behave in delinquent and/or antisocial ways (Delfabbro and King 2012). Consequently males are more likely to reflect such associations than females. A third possibility is associated with the disinhibiting effects of alcohol. Thus it may be that mothers who are more persistent consume of alcohol behave in a more disinhibited manner, and themselves are more likely to gamble and take risks. The gambling behaviour of offspring, in this context, is simply learned behaviour. Another possibility is that mothers who consume more alcohol have offspring who are more likely to consume alcohol (Cleveland et al. 2014; Englund et al. 2008a; Van Der Vorst et al. 2009). The gambling behaviour of offspring may simply reflect their own level of disinhibition which may involve gambling as an outlet. It is, in this study, not possible to determine which of the above possibilities is correct. Specific studies which test some of the above options are needed. However it is appear that we have identified a causal pathway that links maternal alcohol consumption trajectories with levels of alcohol consumption by offspring and offspring problem gambling behaviour.

The present study had some limitations. It is likely that self-reported alcohol consumption typically accounts for up to 60 % of total alcohol sales. Problems with sampling, non-response bias as well as under-reporting bias may all contribute to the underestimates of alcohol consumed (Gmel and Rehm 2004; Greenfield and Kerr 2008; Livingston and Callinan 2015). A self-report measurement of alcohol consumption using ‘*quantities*-*frequency*’ may not be able to completely capture the heaviest drinking group (Armor and Polich 1982). Nevertheless, as noted by researchers, frequency-quantity questionnaires are a generally reliable and valid way of measuring alcohol consumption among populations (Del Boca and Darkes 2003). Future studies should examine group of mothers who involve in long term heavy alcohol consumption in a relation to their offspring’s problem gambling behaviour. The MUSP study has not collected information on father’s alcohol consumption at each phase of the study. We only asked mothers at the 14 year follow-up about father’s alcohol use problems. Therefore, we cannot fully account for the influence of paternal alcohol consumption on offspring gambling. There is a good reason to expect that young adult’s alcohol use or substance use may be related to father’s alcohol consumption (Haugland et al. 2013; Van Der Vorst et al. 2009). It is possible that the association between mother’s alcohol consumption patterns and their young adult’s risk of problem gambling behaviour may partly reflect paternal influences. This could result in residual confounding involving father’s alcohol use levels. More research investigating both the impact of mother and father’s alcohol consumption trajectories on offspring’s problem gambling is needed. Another limitation is that in our study young adult’s gambling behaviour was assessed at the 30 year follow-up, suggesting that it may reflect the prevalence of the gambling status in the late transition period from adolescence to adulthood. Previous studies indicated that gambling behaviours among young adults are more stable than among adolescents (Delfabbro et al. 2014); however, in order to have a comprehensive understanding of the association between maternal alcohol consumption and their offspring’s patterns of problem gambling behaviours, future research should examine the role of maternal alcohol consumption trajectories on their offspring’s gambling behaviour development patterns. Finally, loss to follow-up may have biased some results. Of the cohort of 3416 mothers and their young adult offspring at 21 year follow-up, some 1691 mother-child pairs remained and were eligible for main analyses. The attrition rate was about 50.5 % of the sample. Our multiple imputation analyses indicated that the results are not likely to have been substantially affected by selection bias.

## Conclusion

A cross-gender difference was observed in the associations between maternal drinking trajectories and adolescent problem gambling behaviours. Membership of the maternal moderate-escalating alcohol consumption trajectory independently predicted a risk of problem gambling for male offspring at 30 years—even after adjustment for a range of potential confounding variables. It appears that patterns of maternal alcohol consumption may have an independent effect on their young adult’s risk of problem gambling behaviours, with male children being more vulnerable to the effects of maternal alcohol use than female children. Programs intended to address problem gambling behaviours by young adults may need to focus on male group with a co-occurrence of alcohol consumption and problem gambling as well as alcohol consumption patterns by mothers in addition to other family factors. The findings also suggest that the problems that respondents have with gambling have their origins in the family life and patterns of behaviour of the family of origin of the gambler. This implies that providing services for problem gamblers may be necessary but not sufficient. There is a need to focus on family factors which predict the onset of gambling related behaviour. At one level this raises the need to educate families about gambling before gambling begins. At another level there may be a need to let mothers who consume alcohol be aware of some collateral consequences of such consumption.

## Appendix

**Table:**

Different group models of maternal alcohol consumption trajectory	Parameters of unadjusted trajectory models		
	BIC	Mean posterior probability	N (%)
Two group-model			
Group 1	−23285.23	0.96	1203 (71.1)
Group 2		0.92	488 (28.9)
Three group-model			
Group 1	−22675.65	0.87	277 (16.4)
Group 2		0.93	1092 (64.6)
Group 3		0.92	322 (19.0)
Four group-model			
Group 1	−22771.10	0.83	197 (11.6)
Group 2		0.91	1003 (59.3)
Group 3		0.86	439 (26.0)
Group 4		0.87	52 (3.1)

In the present study, maternal alcohol consumption trajectories were identified by Stata plugin software application. We identified two to four possible group models of maternal alcohol consumption. All models had BIC and posterior probabilities that met the standards generally used. The three drinking trajectory groups was selected as the best fitting model based upon the largest BIC, mean posterior probability, and the size of membership in each trajectory group

## Footnotes

1 For example, if a respondent reported consuming alcohol a few times a week and drinking three to four standard glasses at each time, the mid-point estimate was coded as 3 days a week (for frequency) and 3.5 glasses a day (for quantities consumed). Respondent’s alcohol consumption, in this case, were about 10.5 standard drinks per week as the product of the mid-point estimates of frequency and quantity of alcohol consumption.
